# Blood meal source and mixed blood-feeding influence gut bacterial community composition in *Aedes aegypti*

**DOI:** 10.1186/s13071-021-04579-8

**Published:** 2021-01-28

**Authors:** Ephantus J. Muturi, Teresia M. Njoroge, Christopher Dunlap, Carla E. Cáceres

**Affiliations:** 1 Crop Bioprotection Research Unit, Agricultural Research Service, U.S. Department of Agriculture, 1815 N. University St, Peoria, 61604 IL USA; 2 Department of Entomology, University of Illinois at Urbana-Champaign, 505 S. Goodwin Ave, Urbana, IL 61801 USA; 3 Department of Evolution, Ecology and Behavior, School of Integrative Biology, University of Illinois at Urbana-Champaign, 505 S. Goodwin Ave, Urbana, 61801 IL USA

**Keywords:** Mixed blood-feeding, MiSeq, Gut microbiota, *Aedes aegypti*

## Abstract

**Background:**

The guts of blood-sucking insects host a community of bacteria that can shift dramatically in response to biotic and abiotic factors. Identifying the key factors structuring these microbial communities has important ecological and epidemiological implications.

**Methods:**

We used the yellow fever mosquito, *Aedes aegypti*, to investigate the impact of mixed blood meals on gut microbiota of vector mosquitoes. Adult females were experimentally fed on sugar or blood from chicken, rabbit or a mixture of chicken and rabbit blood, and their gut microbiota were characterized using 16S rRNA gene amplification and MiSeq sequencing.

**Results:**

The gut bacterial communities of mosquitoes fed on the three blood meal treatments clustered separately, suggesting that host species identity and mixed blood-feeding are key determinants of gut bacterial community composition in mosquitoes. Mixed blood meal had a synergistic effect on both operational taxonomic unit (OTU) richness and the Shannon diversity index, suggesting that mixed blood-feeding can offset the nutritional deficit of blood meals from certain host species. The microbial communities observed in this study were distinct from those identified from similarly fed *Ae. aegypti* from our previous study.

**Conclusions:**

These findings demonstrate that vector host-feeding preferences can influence gut microbial composition and diversity, which could potentially impact pathogen acquisition and transmission by the vector. The results also demonstrate that different microenvironmental conditions within the laboratory may play an important role in structuring the microbial communities of independently reared mosquito colonies.

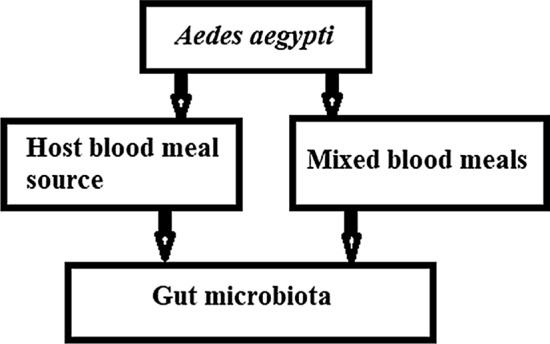

## Background

Blood-feeding arthropods such as ticks, mosquitoes, sand flies and black flies are among the most studied group of animals due to their role in the transmission of pathogens and parasites of medical and economic significance. The majority of arthropod-borne pathogens and parasites infect multiple host species, and due to heterogeneities in host species’ abundance, exposure and susceptibility to infection, certain host species contribute disproportionately to pathogen transmission [1–3]. For example, the intensity and timing of peak West Nile virus (WNV) infection rates in the northern house mosquito, *Culex pipiens pipiens*, is primarily driven by its preference to feed on the American robin, *Turdus migratorius* [4]. Similarly, shifts in tick feeding from the highly infectious white-footed mice, *Peromyscus leucopus*, to other mammalian host species has the potential to reduce the prevalence of Lyme disease in humans and ticks [2]. Therefore, host selection patterns by blood-feeding arthropods are key determinants of epidemiological dynamics for vector-borne pathogens and are typically used to understand the natural transmission dynamics, predict disease risk and inform disease surveillance and control efforts.

An important but overlooked aspect of vector host-feeding preferences that has the potential to alter the dynamics of vector-borne diseases is its impact on arthropods’ gut microbial communities. It is well established that some bacterial species within the arthropod body, especially the gut, enhance vector longevity and reproductive fitness, modulate insecticide resistance and modify vector susceptibility to pathogens [5–13]. Factors that disrupt the composition of these microbial communities may therefore indirectly alter pathogen transmission dynamics by modifying the components of vectorial capacity, including vector competence, daily survivorship, vector density and biting rates. Recent studies have revealed that factors such as arthropod host species, sex, body size, site of collection, stage of development, larval environment, infection with pathogens, genetic background and exposure to xenobiotics can have a strong influence on the gut microbiota of arthropod vectors [14–20]. Host blood-meal source has also been shown to strongly influence the microbial composition and diversity within the arthropod body, but remains an understudied topic [21–23]. Although vector-feeding on multiple host species within the same gonotrophic cycle is fairly common in nature [24–31], the impact of mixed blood meals on vector gut microbial communities remains poorly understood.

Adult females of many mosquito species require a blood meal to obtain essential nutrients for egg maturation. Although different mosquito species are known to preferentially feed on certain vertebrate animal species, mixed blood-feeding within the same gonotrophic cycle is also commonly reported in many mosquito species [24–26, 30, 32–34]. For this reason, many mosquito species provide an excellent model system for studying how mixed blood meals affect the composition and structure of gut microbial communities in arthropod vectors.

In this study, we used the yellow fever mosquito, *Aedes aegypti*, to examine how the gut microbiota of an arthropod vector responds to mixed blood meals. Although this mosquito species is mostly known for its anthropophilic tendency, this behavior can vary markedly across geographic regions, with some populations exhibiting a zoophilic tendency and feeding on multiple vertebrate host species in a single gonotrophic cycle [33–36]. For example, a recent study in western Kenya revealed that only 4% of blood-engorged *Ae. aegypti* had fed on humans, with rock hyraxes, goats, cattle, hippopotamuses, and rock monitor lizards accounting for 79, 9, 4, 1 and 1% of the total blood meals, respectively [35]. Adult females of *Ae. aegypti* were artificially fed on blood from either chicken, rabbit or their mixture and their gut microbiota profiled 7 days post blood meal. This study improves current understanding of the impact of host blood-meal source on microbiome variation in arthropod vectors and provides an opportunity for further studies on how vector host-feeding patterns may affect pathogen acquisition and transmission.

## Methods

### Experimental design

The study was conducted using a laboratory colony of *Ae. aegypti* (Rockefeller strain) that was reared and maintained using our previously described methods [22]. Larvae of this colony are routinely maintained on a diet containing 1:1:1 mixture of rabbit chow, Tetramin fish food and liver powder, and adults are artificially blood-fed on bovine blood. Thirty-six newly emerged adults were sacrificed, and their midguts processed as described below to determine the baseline microbial community composition prior to sugar-feeding and blood-feeding. Other newly emerged adults were housed in plastic cages in batches of approximately 300 individuals and provided access to 10% sucrose solution. Adult females aged 3 to 5 days were sugar-starved for 24 h and provided access to one of the following treatments: continuous supply of 10% sucrose solution, or 1-h access to chicken blood, rabbit blood or an equal mixture of chicken and rabbit blood (Hemostat Laboratories, Dixon, CA, USA). Blood-feeding was accomplished using the artificial blood-feeding system described in Muturi et al. [22]. Blood-fed females from each blood meal treatment were sorted on a chill table, transferred to a new cage and provided continuous access to the 10% sucrose solution. The sucrose solution was replaced every 2 days to prevent fungal growth. At 7 days after blood-feeding, the midguts of sugar-fed and blood-fed adult females were dissected as described below. Overall, midguts were dissected for five treatment groups, including newly emerged adults (NE), sugar-fed adults and adults fed on one of three blood meal types; chicken blood, rabbit blood or a mixture of chicken and rabbit blood (combined).

### DNA extraction, library preparation and sequencing

Mosquito samples were cold anesthetized and surface-sterilized to remove surface bacteria before midgut dissections. Surface-sterilization consisted of a 10-min rinse in a 2% bleach solution, followed by two 5-min rinses in 1× sterile phosphate buffered saline (PBS), a 5-min rinse in 70% ethanol and two rinses in sterile ultrapure distilled water (Invitrogen, Grand Island, NY, USA). Each midgut was dissected on a drop of 1× PBS, and three midgut samples were pooled into sterile microcentrifuge tubes containing 100 μl of sterile PBS. Twelve midgut pools were processed for each treatment for a total of 60 samples. Total DNA from each midgut pool was extracted using the DNeasy Blood and Tissue Kit (Qiagen, Valencia, CA, USA) according to manufacturer’s instructions. Amplification of the V3-V4 region of the 16S rRNA gene was accomplished using the primer set V3-V4F (5′-**TCGTCGGCAGCGTCAGATG-TGTATAAGAGACAG**CCTACGG-GNGGCWGCAG-3′) and V3-V4R (5′-**GTCTCGTGG-GCTCGGAGATGTGTATAAGAGACAG**GACTACHVGGGTATCTAATCC-3′), with the Illumina Nextera overhang adapter sequences (in bold) added to the locus-specific primer sequences as provided in the Illumina 16S metagenomic sequencing library preparation protocol (Illumina, Inc., San Diego, CA, USA). The PCR mixture (25 µl) consisted of 2.5 µl of template DNA (5 ng/µl), 5 µl each of forward and reverse primers (1 µM) and 12.5 µl of 2× KAPA HiFi HotStart ReadyMix (Kapa Biosystems, Roche Holding AG, Basel, Switzerland). Amplification conditions included an initial denaturation at 95 °C, 3 min; then 95 °C/30 s, 55 °C/30 s, 72 °C/30 s for 35 cycles; and a final extension at 72 °C for 5 min. The PCR amplicons were cleaned using AMPure XP beads, and a second PCR was conducted to attach dual indices and Illumina sequencing adapters using the Nextera XT Index Kit. The second PCR was conducted in a 50-µl reaction volume containing 5 µl of DNA from amplicon PCR, 5 µl each of N7 and S5 Nextera XT Index primers, 25 µl of 2× KAPA HiFi HotStart ReadyMix and 10 µl of PCR grade water. Thermocyling conditions for the index PCR were identical to those of amplicon PCR except for the number of cycles (8 cycles vs. 35 cycles for amplicon PCR). A second clean-up was conducted using AMPure XP beads before the PCR amplicons were quantified, pooled and normalized. The pooled library was mixed with Phix control spike-in of 5% as a sequencing control. Sequencing was performed on an Illumina MiSeq system using the MiSeq V3 2 × 300 paired-end sequencing kit. Demultiplexed reads were examined using the CLC genomics workbench v20.0 (Qiagen Inc., Valencia, CA, USA) and low-quality reads and chimeras were removed. The reads were then paired and trimmed to fixed length, and then the CLC Microbial Genomics module was used for operational taxonomic unit (OTU) clustering. The SILVA rRNA gene database project was used to assign OTUs at 97% sequence similarity [37].

### Statistical analyses

R version 3.3.2 (https://cran.r-project.org/bin/windows/base/old/3.2.3/) and the paleontological statistics software package (PAST) version 3.14 [38] were used for statistical analyses. All singletons were discarded before downstream analyses as they may have resulted from sequencing errors [39]. Ten samples that had < 1538 sequences were also excluded from downstream analysis. Alpha and beta diversity analyses were conducted using the entire dataset (considering all sequences) as well as rarefied dataset (1538 reads per sample) to mitigate biases arising from different sampling depths across samples. The ‘vegan’ package in R [40] was used to compute the alpha diversity metrics, including the Shannon diversity index and observed OTUs (richness), and the non-parametric Kruskal–Wallis test was used for statistical comparisons between treatments. Linear contrasts were used to test the mixing of blood for additivity. Mean values for observed OTUs (OTU richness) and the Shannon diversity index in the mixed blood meal treatment were compared to those of the single blood treatments. The effect was classified as additive if the contrast was not significant and synergistic if the effect of blood mixture was significantly greater than the sum of separate effects. Non-metric multidimensional scaling (NMDS) with Bray–Curtis similarity matrix values was conducted using the ‘vegan’ package. NMDS plots to visualize within-group and between-group differences in bacterial communities were generated using the ‘vegan’ package. Stress values were used to assess the quality of NMDS representation; stress values < 0.2 are considered a good representation of the data and those > 0.3 are not considered to be valid [41]. The permutational multivariate analysis of variance (PERMANOVA) test with 9999 permutations was conducted in PAST version 3.14 to determine the statistical significance. Indicator species analysis was conducted using the ‘indicspecies’ package to determine the bacterial genera that were strongly associated with each treatment [42]. Rarefaction curves to determine the sequencing coverage for each treatment were generated using the ‘vegan’ package.

## Results

**Fig. 1:**
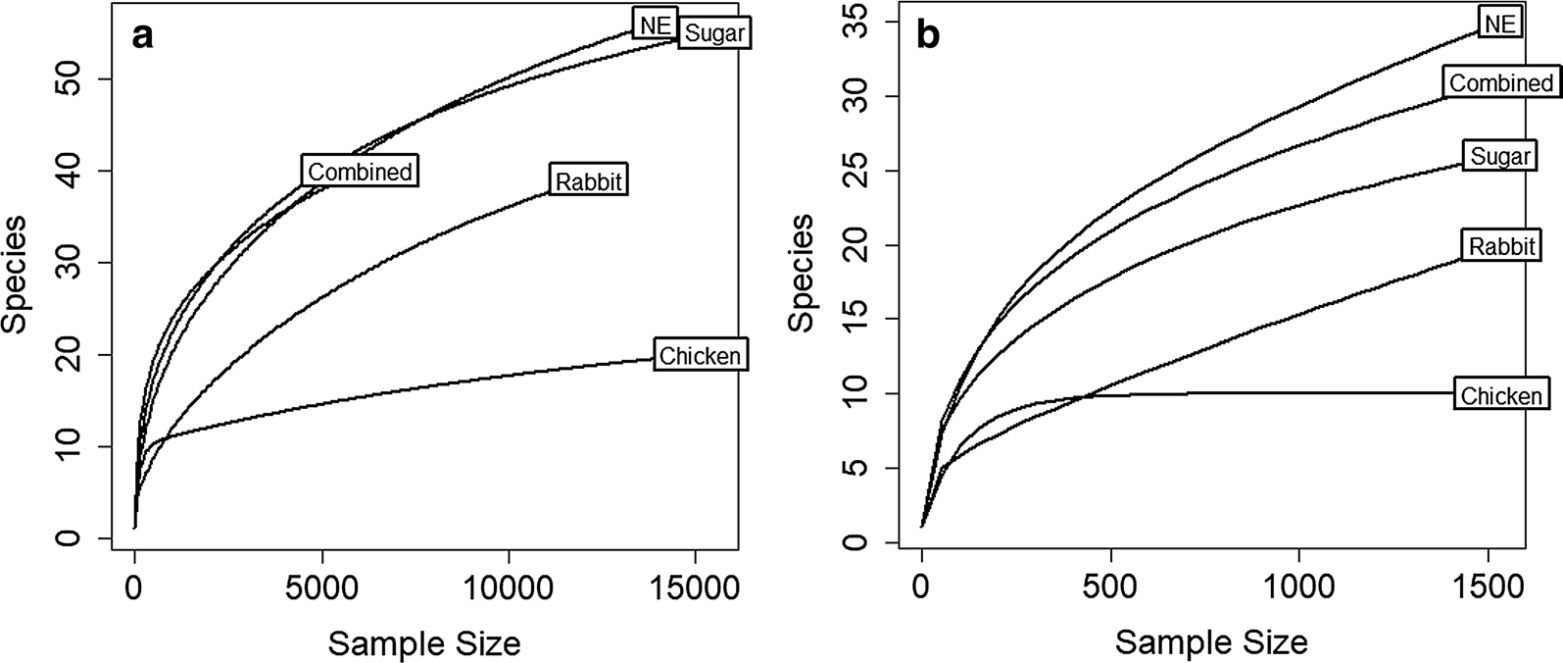
Rarefaction curves for mosquitoes from different treatments generated using (**a**) the entire dataset and (**b**) the rarefied dataset

**Fig. 2:**
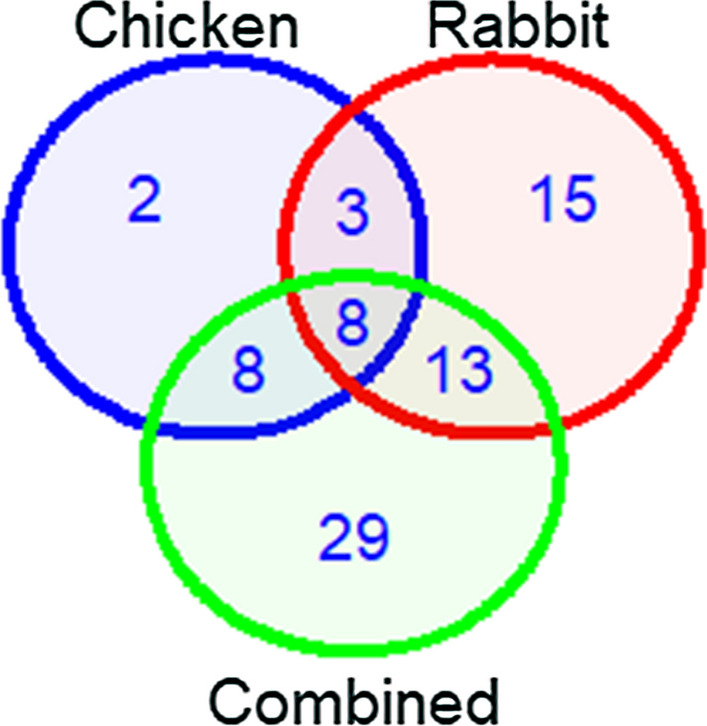
Venn diagram summarizing the overlap of bacterial operational taxonomic units among mosquitoes fed on chicken blood, rabbit blood or a mixture of chicken and rabbit blood (Combined)

After merging the paired end reads, MiSeq sequencing of the 16S rDNA amplicon generated 635,605 sequences from 60 pools of mosquito samples. Reads per pool of mosquito samples ranged from 41 to 69,568 sequences. Rarefaction curves for the entire dataset and the rarefied dataset indicated that the microbial communities of mosquitoes fed on chicken blood were adequately recovered, but the curves for the remaining treatments did not reach a plateau, suggesting that most but not all microbiota were detected (Fig. 1). Fifty mosquito pool samples were retained for downstream analysis after quality filtering and rarefaction to an even depth of 1538 sequences per sample. Overall, a total of 113 OTUs belonging to 46 genera were identified at the 97% cutoff threshold. The total number of OTUs identified by sample type were 66 OTUs in newly emerged adults, 58 OTUs in sugar-fed mosquitoes, 21 OTUs in mosquitoes fed on chicken blood, 39 OTUs in mosquitoes fed on rabbit blood and 58 OTUs in mosquitoes fed on a mixture of chicken and rabbit blood. Venn diagram comparing the overlap of OTUs between mosquitoes fed on chicken blood, rabbit blood and a mixture of chicken and rabbit blood revealed eight OTUs that were shared among the three treatments and two, 15 and 29 OTUs that were unique to mosquitoes fed on chicken blood, rabbit blood and a mixture of chicken and rabbit blood, respectively (Fig. 2).

Significant treatment differences in bacterial OTU richness and diversity were observed (Table 1). Bacterial OTU richness was significantly higher in mosquito samples fed on mixed blood meals than in those fed on chicken blood alone (Kruskal–Wallis test:* χ*^2^ = 18.011,* df* = 4, *P* = 0.0012). There was no significant difference in OTU richness between newly emerged mosquitoes and mosquitoes fed on sugar, chicken blood or rabbit blood. Similarly, OTU richness was not significantly different between newly emerged mosquitoes and mosquitoes fed on sugar, rabbit blood or a mixture of rabbit and chicken blood. Shannon diversity index indicated that the microbial communities of mosquitoes fed on either sugar, rabbit blood or a mixture of chicken and rabbit blood were significantly more diverse than those of mosquitoes fed on chicken blood (Kruskal–Wallis test: * χ*^2^ = 32.087, *df =* 4, *P* < 0.0001). Mosquitoes fed on sugar and rabbit blood also had significantly higher Shannon diversity index compared to newly emerged mosquitoes. The test for additivity revealed a synergistic effect of blood meal mixture on both OTU richness (*t* = − 5.073, *df =* 16.95, *P* < 0.00001) and Shannon diversity index (*t * = − 2.535, *df =* 26.25, *P* = 0.0176). Alpha diversity results for the entire dataset were mostly identical to those for the rarefied dataset, thereby confirming that rarefaction did not lead to a loss of information (Table 1).

**Table 1:** Diversity of bacterial communities in the midguts of adult females of *Aedes aegypti*

Treatment	No. of mosquito pools (initial)	No. of mosquito pools (final)	Rarefied data (1538 sequences)		All data		
			OTU richness	Shannon diversity	OTU richness	Shannon diversity	Total sequences
Chicken	12	10	11.70 ± 0.33a	0.50 ± 0.02a	17.60 ± 0.58	0.50 ± 0.02	15,165.1 ± 814.29
Combined	12	12	17.58 ± 0.91b	1.26 ± 0.11bc	25.08 ± 1.38	1.26 ± 0.11	6038.75 ± 923.05
Newly emerged adults	12	10	14.20 ± 1.91b	0.71 ± 0.13ac	20.30 ± 1.66	0.71 ± 0.09	13,945 ± 6727.96
Rabbit	12	8	13.25 ± 0.92b	1.35 ± 0.05b	22.38 ± 1.83	1.36 ± 0.05	12,147.38 ± 4739.73
Sugar	12	10	16.50 ± 1.48ab	1.55 ± 0.08b	26.44 ± 2.36	1.55 ± 0.08	15,530.6 ± 5066.28

Values are presented as the mean ± standard error (SE) unless indicated otherwise. Statistical analysis was conducted for rarefied data only. Values with different lower case values within a column are significantly different at *P* ≤ 0.05

There were three individuals per pool

**Fig. 3:**
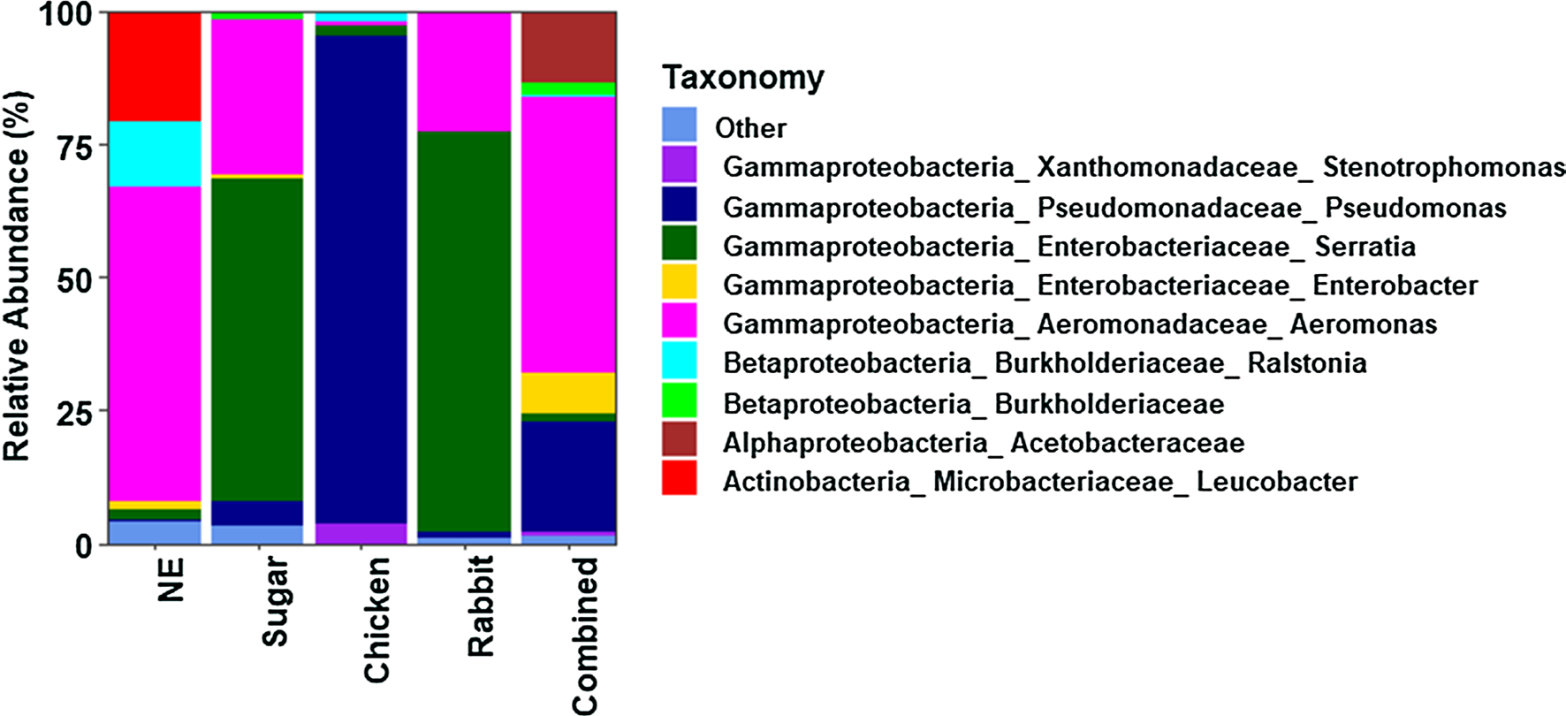
Relative abundances of bacterial taxa associated with newly emerged mosquitoes (*NE*), sugar-fed mosquitoes and mosquitoes fed on chicken blood, rabbit blood or a mixture of chicken and rabbit blood (Combined)

The top nine genera accounted for 96.12 and 96.86% of the total sequences in newly emerged adults and sugar-fed adults, respectively, compared to 99.97, 99.14 and 98.70% of the total sequences in adults fed on blood from chicken, rabbit and chicken–rabbit blood mixture, respectively. The relative abundance of these genera varied markedly across treatments (Fig. 3). The five most abundant genera in newly emerged adults were *Aeromonas* spp. (59.09%), *Leucobacter* spp. (20.81%), *Ralstonia* spp. (12.11%), *Serratia* spp. (1.80%) and *Enterobacter* spp. (1.68%), collectively accounting for 95.48% of the total sequences. In sugar-fed adults, the five most abundant genera accounted for 96.55% of the total sequences and included *Serratia* spp. (60.83%), *Aeromonas* spp. (29.25%), *Pseudomonas* spp. (4.39%), unclassified Burkholderiaceae (1.63%) and *Enterobacter* spp. (0.45%). In mosquitoes fed on chicken blood, the topmost abundant bacterial genera were *Pseudomonas* spp. (91.43%), *Stenotrophomonas* spp. (3.75%), *Ralstonia* spp. (2.12%), *Serratia* spp. (1.97%) and *Aeromonas* spp. (0.69%), collectively accounting for 99.95% of the total sequences. *Serratia* spp. (75.15%), *Aeromonas* spp. (22.83%), *Pseudomonas* spp. (1.13%) and unclassified Burkholderiaceae (0.03%) were the most abundant genera, accounting for 99.14% of the total sequences. Mosquitoes fed on a mixture of chicken and rabbit blood were dominated by *Aeromonas* spp. (51.74%), *Pseudomonas* spp. (20.72%), unclassified Acetobacteraceae (13.36%), *Enterobacter* spp. (7.68%) and unclassified Burkholderiaceae (2.39%), collectively accounting for 95.88% of the total sequences. The results for the whole data set were identical to those for the rarefied data (Additional file 1: Fig. S1).

Indicator species analysis identified some bacterial taxa that were significantly associated with specific treatment groups (Table 2). *Leucobacter* spp. and *Sphingobacterium* spp. were significantly associated with newly emerged adults while *Pseudomonas* spp. and *Stenotrophomonas* spp. were significantly associated with mosquitoes that fed on chicken blood. *Rahnella* spp. and *Actinotignum* spp. were significant indicator species for mosquitoes fed on rabbit blood, while unclassified Acetobacteraceae was a significant indicator species for mosquitoes fed on a mixture of chicken and rabbit blood. A few bacterial taxa, including *Aeromona*s spp., *Serratia* spp., Burkholderiaceae and Enterobacteriaceae, were significantly associated with more than one treatment group (Table 2). No indicator species were identified for sugar-fed mosquitoes.

**Table 2:** Indicator species for adult *Ae. aegypti* fed on different diet types

Treatment	Bacterial taxa	Indicator value	*P* value
Newly emerged adults	*Leucobacter* spp.	0.476	0.0036
	*Sphingobacterium* spp.	0.372	0.0401
Chicken blood	*Pseudomonas* spp.	0.967	< 0.001
	*Stenotrophomonas* spp.	0.930	< 0.001
Rabbit blood	*Rahnella* spp.	0.436	0.0307
	*Actinotignum* spp.	0.434	0.0239
Combined	Acetobacteraceae	0.538	< 0.001
Newly emerged mosquitoes + Combined	*Aeromona*s spp.	0.619	< 0.001
Sugar + Combined	Burkholderiaceae	0.641	< 0.001
Sugar + Rabbit	*Serratia* spp.	0.913	< 0.001
	Enterobacteriaceae	0.624	< 0.001

Analysis was performed using the rarefied data set

**Fig. 4:**
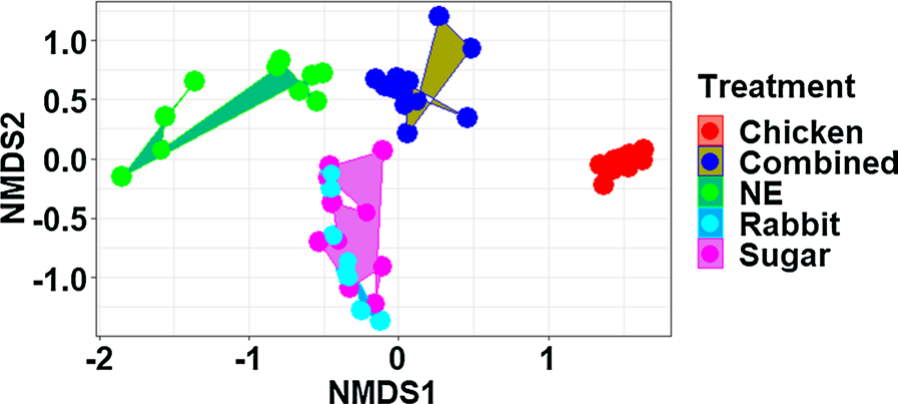
Non-metric multidimensional scaling (*NMDS*) ordination of Bray–Curtis distances between bacterial communities from newly emerged mosquitoes (*NE*), sugar-fed mosquitoes and from mosquitoes fed on chicken blood, rabbit blood or a mixture of chicken and rabbit blood (Combined)

NMDS analysis using the rarefied dataset (Stress = 0.128; Fig. 4) and the entire dataset (Stress = 0.152; Additional file 2: Fig. S2) revealed a strong effect of treatment on the gut bacterial communities of *Ae. aegypti*. Bacterial communities within each treatment clustered together and were more similar than bacterial communities between treatments. The only exception to this observation was between bacterial communities of sugar-fed mosquitoes and mosquitoes fed on rabbit blood, which clustered together. PERMANOVA analyses confirmed that the observed differences in microbial communities between sample types were statistically significant with exception of comparisons between sugar-fed mosquitoes and mosquitoes fed on rabbit blood (*F* = 27, *P* = 0.0001; Table 3).

**Table 3:** Permutational multivariate analysis of variance results comparing treatment differences in microbial communities

Treatments	Chicken	Combined	Newly emerged mosquitoes	Rabbit
Chicken				
Combined	60.64			
Newly emerged adults	36.61	5.963		
Rabbit	178.3	33.11	13.47	
Sugar	111.4	23.54	11.77	1.272

All pairwise comparisons were significant at *P* < 0.01 after Bonferroni corrections for multiple comparison, with the exception of comparisons between sugar-fed mosquitoes and mosquitoes fed on rabbit blood

## Discussion

Although it is well known that disease vectors can blood-feed on multiple vertebrate hosts within the same gonotrophic cycle [24–31] and that the blood meal source can have a strong impact on vector microbiota [21–23], the impact of mixed blood meals on vector microbiota remains poorly understood. This study focused on how mixed blood meals affect the dynamics of gut microbiota in mosquitoes. We specifically focused on the impact of the chicken and rabbit blood mixture based on our previous findings that blood meals from the two vertebrate host species have variable effects on gut microbiota of *Ae. aegypti* [22]. Although this simplified model system may not be a perfect representation of the complex system that occurs in nature, it highlights the need for further studies to decipher the ecological and epidemiological impact of mixed blood feeding by different mosquito species and other hematophagous arthropods. Overall, our results show that blood meal source and mixed blood-feeding are key factors influencing the dynamics of gut microbiota in *Ae. aegypti* and likely other mosquito species also.

Blood meal source had a significant impact on microbial community composition and structure. Microbial communities of mosquitoes fed on blood from chicken, rabbit or their mixture clustered separately from each other and from those of newly emerged adults. The distance separating the microbial communities of mosquitoes fed on chicken versus rabbit blood was greater than the distance separating the microbial communities of mosquito samples fed on single-host blood meals (i.e. chicken or rabbit blood) versus mosquito samples fed on a mixture of chicken and rabbit blood. Compared to chicken blood, mammalian blood has higher amounts of hemoglobin, hematocrit and total protein [43], and mixing the two blood types may narrow the compositional differences. However, additional studies are needed to examine how mixing blood from different host species affects the nutritional composition and quality of the blood meal.

Mixing chicken and rabbit blood likely increased the diversity of distinct molecules available for utilization by some specialist bacteria that were better adapted to utilize resources from either chicken or rabbit blood meals as well as some generalist bacteria that were able to consume and metabolize the diverse molecules available in mixed blood meals. For example, *Pseudomonas* spp. and *Serratia* spp. were the dominant bacterial taxa in mosquito samples fed on chicken and rabbit blood meals, respectively, but they occurred in much lower abundance in mosquitoes fed on mixed blood meals. In contrast, *Aeromonas* spp., unclassified Acetobacteraceae, *Enterobacter* spp. and unclassified Burkholderiaceae were more abundant in mosquitoes fed on mixed blood meals and were either less abundant or absent in mosquitoes fed on chicken or rabbit blood meals. Suppression of *Pseudomonas* spp. and *Serratia* spp. in mosquitoes fed on mixed blood meals may therefore have created an open niche that was exploited by *Aeromonas* spp., unclassified Acetobacteraceae, *Enterobacter* spp. and unclassified Burkholderiaceae. Additionally, we detected 29 OTUs that were unique to mosquitoes fed on mixed blood meals, suggesting that mixed blood meals supported some bacterial taxa whose growth was limited in chicken and rabbit blood meals, respectively. The hydrolysis of hemoglobin during blood meal digestion is also known to produce large amounts of toxic oxidants, and some bacterial taxa are better adapted to cope with oxidative stress than others [15, 44, 45]. It is possible that the three blood meal treatments yielded different amounts of toxic oxidants given their expected differences in hemoglobin content, leading to differential elimination of some bacterial taxa. However, the impact of oxidative stress on bacterial communities was not investigated in this study.

It has long been established that resource concentration and composition are key determinants of species diversity and food web complexity, with the more concentrated and diverse resource supporting higher species’ diversity and productivity [46–49]. Some bacterial taxa are highly specialized in terms of their carbon use, and thus resource heterogeneity may promote microbial diversity through resource niche partitioning [49, 50]. There was no strong evidence that mixed blood meals promoted higher bacterial diversity and richness compared to blood meals from a single host species; gut bacterial diversity and richness in mosquitoes fed on mixed blood meals were not significantly different from those of mosquitoes fed on rabbit blood. However, mosquitoes fed on chicken blood had a significantly lower bacterial diversity, as shown by a significantly lower Shannon diversity index, than mosquitoes fed on blood from rabbit or a mixture of chicken and rabbit blood, and the test for additivity revealed a synergistic effect of blood mixture on both OTU richness and diversity. These findings suggest that chicken blood meal is a low-quality resource for some gut bacterial communities and that mixed blood meals from some vertebrate host species may offset this nutritional deficit.

Mosquitoes fed on sucrose and rabbit blood formed a single cluster that was separate from mosquitoes fed on chicken blood or a mixture of chicken and rabbit blood. We did not examine the mechanism(s) underlying this observation but can offer two possible explanations. First, the results may indicate that chicken blood and a mixture of chicken and rabbit blood cause long-term changes in gut microbial communities of the vector while rabbit blood causes transient changes in microbial communities that are later succeeded by sugar-utilizing bacteria once the blood meal is digested. Second, it is also possible that rabbit blood and sucrose supported similar bacterial communities. Additional studies capturing temporal changes in microbial communities in sugar-fed and blood-fed mosquitoes could be more revealing.

Newly emerged mosquitoes and mosquitoes fed on chicken blood had significantly lower bacterial diversity (Shannon diversity index) compared with sugar-fed mosquitoes and mosquitoes fed on rabbit blood. In our previous study using the same mosquito species (*Ae. aegypti*), the gut microbial communities of newly emerged adults and adults profiled 7 days after feeding on human blood were significantly more diverse than those of sugar-fed mosquitoes and mosquitoes fed on chicken, rabbit or human blood and profiled 3 days post blood meal [22]. A previous study by Wang et al. [15] revealed that a blood meal from the U.S. National Institutes of Health (NIH) Swiss outbred mice drastically reduced the diversity of the gut bacterial community and favored enteric bacteria in *Anopheles gambiae*. These findings suggest that the impact of sugar-feeding and blood-feeding on mosquito microbiota is complex and can vary markedly even for the same mosquito species depending on the underlying microenvironmental conditions. Additional studies targeting multiple mosquito species and using multiple host blood-meal sources would provide more insights into how adult mosquito diet affects the diversity of gut bacterial communities.

The gut microbial communities observed in this study differ from those reported in our previous study in which we evaluated the impact of host blood-meal source on gut microbiota of *Ae. aegypti* [22]. We had expected the microbial communities from the two studies to be similar based on our previous findings that diverse laboratory colonies of *Ae. aegypti* reared in the same insectary harbor similar gut bacterial communities [51]. However, the two studies were conducted at different times of the year and had different experimental designs which may have accounted for the observed differences in microbial composition. In the current study, newly emerged adults were provided access to 10% sucrose for 3–5 days, sugar-starved for 24 h before blood-feeding and then dissected 7 days post blood meal. In contrast, newly emerged adults in our previous study were sugar-starved for 3 days before being sugar-fed or blood-fed, and dissections were done 3 days post blood meal [22]. Differences in experimental design and the timing of the two studies may have presented different microenvironments that impacted the type of microbes that colonized and thrived in the insectary. A recent study by Saab et al. [52] found that different cohorts of mosquitoes reared under identical laboratory conditions differed in their microbial composition due to changes in the microenvironment of the insectary attributed to environmental microbes introduced by human activity.

Changes in the composition and abundance of gut microbiota of the vector in response to mixed blood meals has the potential to alter the transmission dynamics of vector-borne pathogens. Some of the microbial taxa disrupted by mixed blood meals, such as *Pseudomonas* spp., *Serratia* spp., *Enterobacter* spp. and *Aeromonas* spp., have been shown to affect vector susceptibility to parasites and pathogens. Isolates of *Pseudomonas rhodesiae* and *Enterobacter ludwigii* from the digestive tract of *Ae. albopictus* have been reported to inhibit La Crosse virus* in vitro* [53], and in another study the abundance of Enterobacteriaceae was greater in the midgut of *Anopheles gambiae* infected with *Plasmodium falciparum* [19]. *Serratia marcescens* was observed to inhibit *Plasmodium* development within the midgut of *Anopheles stephensi* [54, 55] and secreted SmEnhancin protein that rendered *Ae. aegypti* highly susceptible to dengue virus [56]. *Serratia odorifera* and *Aeromonas culicicola* were found to enhance the susceptibility of *Ae. aegypti* to dengue virus [5, 57]. *Anopheles albimanus* Weidemann coinfected with *Serratia marcescens*, *Enterobacter cloacae* and *Enterobacter amnigenus* had significantly lower *Plasmodium vivax* infections compared with the controls, and mosquitoes coinfected with *En. cloacae* had lower oocyst density compared with the controls [58]. These findings suggest that shifts in gut microbiota in response to mixed blood meals have the potential to alter vector susceptibility to pathogens/parasites but that the direction of impact may vary across mosquito-borne disease systems. Thus, studies that examine how shifts in gut microbial communities of the vector in response to mixed blood meals affect mosquito life-history traits, such as fecundity, fertility, longevity and vector competence for different pathogens/parasites, are needed to provide a comprehensive understanding of the ecological and epidemiological implications of mixed blood meals on different mosquito-borne disease systems. Such studies may lead to the discovery of novel bacterial species that may limit transmission of vector-borne diseases either by inhibiting pathogen development within the vector or by suppressing vector populations through effects on longevity, fecundity or fertility. Previous studies have shown that host blood-meal source can have strong effects on mosquito fecundity, fertility and longevity [59–61], suggesting the potential for mixed blood meals to produce antagonistic, additive or synergistic effects on epidemiologically relevant life-history traits of the vector.

## Conclusions

The results of this study show that mixed blood-feeding may alter the gut microbial composition in *Ae. aegypti* and may enhance the diversity of these microbes by offsetting the nutritional deficits of a low-quality host blood meal. Further studies are needed to determine how shifts in midgut bacterial communities in response to mixed blood-feeding affects the components of vectorial capacity. Additionally, future studies should examine how mixed blood feeding is likely to influence the bacterial taxa that are currently under investigation for potential application in symbiotic control of mosquito-borne diseases, such as *Wolbachia* spp. and *Asaia* spp.

## Supplementary information

**Additional file 1: Figure S1.** Relative abundances of bacterial taxa associated with newly emerged mosquitoes (*NE*), sugar-fed mosquitoes, and mosquito fed on chicken blood, rabbit blood or a mixture of chicken and rabbit blood (Combined). The analysis was conducted using the entire dataset.

**Additional file 2: Figure S2.** Non-metric multidimensional scaling (*NMDS*) ordination of Bray–Curtis distances between bacterial communities from newly emerged mosquitoes (*NE*), sugar-fed mosquitoes, and mosquito fed on chicken blood, rabbit blood or a mixture of chicken and rabbit blood (combined). The analysis was conducted using entire dataset.

## Data Availability

All relevant data are either within the paper and its additional files or will be submitted in a public repository at National Center for Biotechnology Information (NCBI) upon acceptance of the manuscript.

## Abbreviations

NMDS: Non-metric multidimensional scaling
NSF: National Science Foundation
OTU: Operational taxonomic unit
PERMANOVA: Permutational multivariate analysis of variance
USDA: United States Department of Agriculture
WNV: West Nile virus
USDA: United States Department of Agriculture
