# Supplementary figures and images for: Blood meal source and mixed blood-feeding influence gut bacterial community composition in Aedes aegypti
Source: Parasit Vectors. 2021 Jan 28;14:83. doi: 10.1186/s13071-021-04579-8 (PMC7841894; doi:10.1186/s13071-021-04579-8)

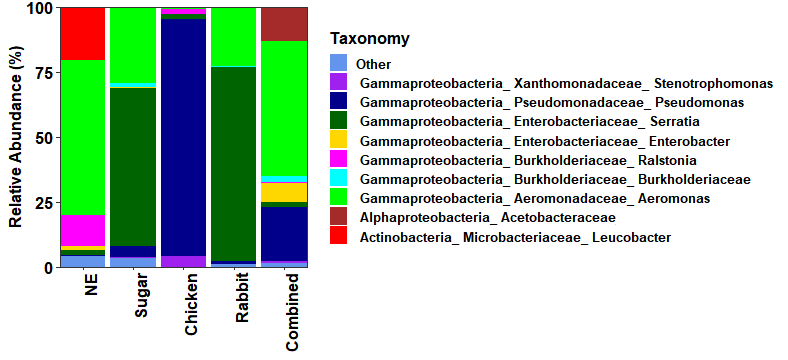

Supplement: Supplementary file 1 — Additional file 1: Figure S1. Relative abundances of bacterial taxa associated with newly emerged mosquitoes (NE), sugar-fed mosquitoes, and mosquito fed on chicken blood, rabbit blood or a mixture of chicken and rabbit blood (Combined). The analysis was conducted using the entire dataset. [file 13071_2021_4579_MOESM1_ESM.tiff]

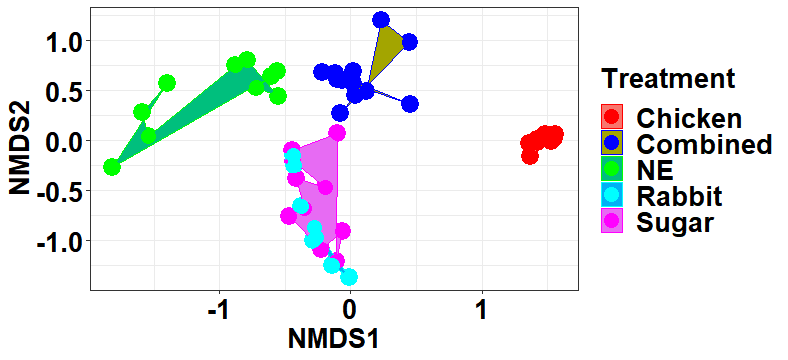

Supplement: Supplementary file 2 — Additional file 2: Figure S2. Non-metric multidimensional scaling (NMDS) ordination of Bray–Curtis distances between bacterial communities from newly emerged mosquitoes (NE), sugar-fed mosquitoes, and mosquito fed on chicken blood, rabbit blood or a mixture of chicken and rabbit blood (combined). The analysis was conducted using entire dataset. [file 13071_2021_4579_MOESM2_ESM.tiff]
